# Generative artificial intelligence for general practice; new potential ahead, but are we ready?

**DOI:** 10.1080/13814788.2025.2511645

**Published:** 2025-06-06

**Authors:** Geert-Jan Geersing, Niek J. de Wit, Matthew Thompson

**Affiliations:** ^a^ Julius Center for Health Sciences and Primary Care, Department of General Practice & Nursing Science, University Medical Center Utrecht, Utrecht University, Utrecht, The Netherlands; ^b^ Department of Family Medicine, University of Washington, Seattle, WA, USA

**Keywords:** Generative AI, clinical decision support, digital innovation

## Abstract

**Background:**

Generative AI (Gen AI) is frequently cited as an innovation to address the current challenges in healthcare, also for primary care. Examples include automating tasks like voice-to-notes transcription or chatbots using large language models. Additionally, it may facilitate a learning healthcare system by generating personalised learning resources and real-time literature summaries. Yet – probably with the highest expectations – Gen AI may extend diagnostic and therapeutic capabilities in general practice by integrating complex, multimodal patient data for personalised care, enabling earlier disease detection, and providing real-time guidance for diagnostics, prognostics and treatments.

**Method & discussion:**

The authors of this opinion paper recently hosted a workshop at the WONCA Europe 2024 conference. From discussions at that workshop, three priorities emerge: practice support, education support, and clinical decision-making support. In this opinion paper, we argue that GPs and academic departments of primary care should lead in evaluating Gen AI across these three priorities. Primary care research must prioritise rigorous scientific evaluations, to ensure that developed tools actually work for GPs and their patients.

**Conclusion:**

Hereto, a coordinated effort, driven by the primary care academic community, is needed, starting with research agenda drafting. A broad, international follow-up is scheduled following this WONCA Europe 2024 workshop.

## KEY MESSAGES

Generative AI is expected to have a broad impact on healthcare, including general practice.

Innovation is likely expected across three areas: practice support, education support, and clinical decision-making support.

A coordinated effort is urgently needed to engage the international primary care academic community to draft a research agenda alongside these three areas.

Since its first inception in the late 80s and early 90s, the development of artificial intelligence (AI) has progressed rapidly. The current phase of Generative AI (Gen AI) models has spurned enormous investment from major technology companies. Gen AI is an extension to existing AI methodology largely driven by large language models (LLMs). In contrast to traditional AI predictive analytics like machine learning or advanced regression models, it has the potential to generate new data. This includes not only text, but also other types of data such as images, video, audio, etc. Gen AI is widely predicted to have a major impact in society in terms of how humans interact with (generated or ‘real-world’) data.

Gen AI is also attracting attention as a possible solution to many of the current challenges that healthcare faces, by supporting process efficiency, quality-of-care, and (ultimately) clinical decision making. The authors recently hosted an international workshop at the WONCA Europe 2024 conference in Dublin. The aims of this workshop were i) to learn from each other in terms of how primary care-based research networks are orchestrated in different countries in relation to AI potential ii) to make a start with drafting a research agenda for AI and primary care research; and finally iii) to start building a consortium of dedicated primary care researchers in this field. We used both small group discussions as well as a moderated discussion. The workshop was well-attended, and a list on enthusiastic delegates for consortium building was drafted. Based on these discussions during that workshop, we will outline the potential applications of Gen AI for primary care in Europe and specifically focus on the research agenda to support implementation.

## Where can Generative AI support general practice?

GPs in most countries in Europe feel overburdened by administrative tasks, many of which have ballooned over recent years. Therefore, as a first example where Gen AI can support primary care, reducing administrative burden is a key area where it can make immediate impact, with goals of improving efficiency of care, reducing GP overload and preventing burnout. Gen AI-based administrative support tools – based upon LLM-technology – such as ‘voice-to-notes’ systems, and applications that can summarise patient records and create referral letters or other documents, are already being used to reduce documentation burden and free up time for patient related tasks [1,2]. Also, traditional AI tools already for many years were able to automatically calculate various disease-related risk scores which could guide drug prescription or referral, for example clinical decision rules for ruling-out venous thrombo-embolism or predicting bleeding risk for patients on anticoagulant therapy [3]. Many of these risk scores are presently burdensome to populate within the medical record in ‘real-time’. Similarly, Gen AI based symptom checkers or triage tools could provide detailed and individualised support for ‘front office’ triage and improve the quality of patient selection and urgency of consultation; all areas that may help reducing the current administrative burden in primary care.

Second, Gen AI can also potentially support training and education [4]. Primary care trainees (and practising clinicians) often struggle to find ‘just in time’ information to support their learning needs. Here, Gen AI can rapidly find or produce digital summaries of scientific literature (and even translated versions of these into lay language to share with patients). Increasingly, Gen AI can generate several aspects of critical appraisal or health information that formerly took too much time for the busy clinician. In systems where GPs take part in regular appraisal or practice evaluation, Gen AI could potentially provide rapid digital individual feedback to GPs and trainees based on scientific literature, progress evaluation and peer reflection. It thereby contributes to practice-based learning [5]. Over time, Gen AI can potentially also create data-driven learning plans and collate bespoke learning resources for trainees [6,7].

Third, likely in the near future, Gen AI can also be instrumental in extending the diagnostic capabilities in primary care, which has been noted as a limitation in many countries. Many GPs are familiar with the interpretation that modern 12-lead ECG devices provide automatically, already using traditional AI techniques [8]. Gen AI can potentially extend the real-time interpretation to other types of tests (where these are available in the GP’s office or nearby). Examples include laboratory tests [9], pulmonary function tests, and even provide quick interpretation of imaging tests that are either performed in general practice (e.g. ultrasound) or in hospitals (X-ray, CT, MRI). Access to imaging is often cited as a major barrier to general practice and extending access to these types of diagnostics could dramatically extend and improve quality of GP services [10].

Fourth, and most importantly, Gen AI can help to improve the quality of clinical decision-making in general practice. We elaborate on this topic in more detail below.

## Generative AI and its role in clinical decision-making in general practice

Clinical decision-making (CDM) refers to the process by which clinical decisions are made at the patient level spanning diagnosis, prognosis and therapeutic interventions. Attendees at the WONCA workshop highlighted multiple areas of CDM where they would value Gen AI support. These areas are summarised below with some examples depicted in Table 1 and a description on how Gen AI may potentially help in future patient care in Box 1.

**Table 1.** Clinical examples where AI may be of value for the purpose of clinical decision making in primary care.

Clinical problem	Clinical dilemma and potential role for AI	Refs^a^
*Prognostic assessment*		
Stroke risk in patients with AF	AF is a highly heterogeneous condition with stroke risk varying based upon underlying clinical determinants, biomarkers, and AF morphology; Gen AI may help in patient stratification and tailor treatment accordingly.	[11]
Hospitalisation risk in LRTI	LRTIs are a frequent reason for encounter in general practice and decisions to refer for hospitalisation are not always straightforward; Gen AI may help selecting patients in need for referral based upon all available EHR data.	[12]
*Diagnostic assessment*		
Sepsis recognition	Sepsis remains an important and easily missed condition with a high mortality risk, also (or notably) in primary care. Gen AI models may provide early warning signals to select patients with infectious diseases at highest risk of sepsis.	[13]
Assessment in suspected PE	Assessment of patients with suspected PE is difficult, certainly in primary care; Gen AI based models may help in predicting the actual risk of PE in each patient.	[14]
*Therapeutic interventions*		
Drug selection in T2DM	The number of antidiabetic drugs has grown enormously over recent years making it difficult to select optimal treatment regimens in primary care; Gen AI may help in suggesting drug combinations based upon guidelines and EHR data.	[15]
HF monitoring	Notably frail elderly patients with HF are at high risk of (re-)hospitalisation. Multimodal data and Gen AI may help busy primary care clinicians, to monitor signs, symptoms and fluid status remotely, e.g. after a hospitalisation.	[16]
*Unravelling new insights*		
Detection of pancreatic cancer	For cancers where there is no routine screening recommended, such as pancreatic cancer, Gen AI tools may identify new patterns in patient consultations, laboratory abnormalities or changes in body weight in primary care that subsequently could trigger diagnostic suspicion and targeted diagnostic work up in selected patients.	[17]
Surveillance of infections	During an unfolding epidemic, patients first seek contact with their GP. Primary care-based surveillance data (using EHR) and a Gen AI model may identify this unfolding epidemic/pandemic earlier than hospital-based data as patients inevitably are first ‘picked up’ in general practice.	[18]

AF: Atrial fibrillation; T2DM: Type 2 diabetes; LRTI: Lower respiratory tract infection; PE: Pulmonary embolism; EHR: Electronic healthcare records; HF: Heart failure; GP: General practitioner.

^a^ Selected references for further reading on this topic.

- For *diagnostic assessment,* Gen AI could provide quantitative support to real-time decision-making for diagnostic decisions, considering clinical guidelines and country- and setting specific cut-off points and retrieving (often ‘hidden’) information from the individual patient record. This could include providing real time quantitative guidance on for example whether C-reactive protein should be measured in a patient with a lower respiratory tract infection to tailor antibiotic treatment accordingly. Other examples include e.g. the interpretation of D-dimer testing to rule-out suspected pulmonary embolism or assess the need for referral for diagnostic work-up in cases of increased risk of lung cancer, as was recently demonstrated [19].
- An important use case is *prognostic assessment* of the risk of disease progression or, alternatively, favourable outcome. Gen AI can help in assessment of this ‘prior’ risk and subsequently tailor this risk by quickly integrating all the information available, such as medical history, existing risk factors, signs and symptoms and family history. This can then result in an individualised risk profile for a disease or outcome, but also in a risk profile for a spectrum of different outcome scenarios.
- Gen AI can also support decision-making for therapeutic interventions. Treatment regimens have become increasingly more complex. For example: atrial fibrillation used to be seen as one single condition ‘only’, requiring anticoagulation for stroke prevention. Currently there is increasing evidence that different subtypes of atrial fibrillation exist, depending on underlying atrial and electrophysiological substrates, related to different patterns of paroxysms, stroke risk or other cardiovascular complications (notably heart failure) [20,21]. Similarly, where diabetes management used to be relatively simple, with limited treatment options (basically ‘only’ sulphonylureas, metformin, or insulin), there is increasing evidence that additional subtypes of diabetes now exist [22]. Moreover, the number of antidiabetic drugs – including all the different options to combine these drugs – has seen a staggering increase. Facing this complexity of therapeutic selection, the need for guideline adherence, and increasing costs, Gen AI could potentially bring together the existing scientific knowledge on the efficacy of different treatment options and weigh them in the context of the individual patient. Gen AI could take into account characteristics, disease history, and symptoms, and generate a real-time overview of pros and cons of options for an individual patient. This would be the starting point of more sophisticated shared decision making in general practice.
- Finally, Gen AI based upon LLM-technology can unravel previously unknown information to further improve clinical management. For example, LLM-based analyses of large databases with primary care patient files were shown to identify patients with a fourfold increased cancer risk, importantly four months before they were actually diagnosed with lung cancer [19,23]. Potentially, such earlier cancer detection might then improve cancer prognosis, certainly for cancer types that are known for their late clinical presentation and poor prognosis, such as lung, ovarian or pancreatic.

Box 1. Illustrative patient example of the potential role of Generative AI in CDM

Mrs. Sparrow is a 73-year-old woman visiting her GP with lower abdominal complaints, a slightly elevated temperature and bloody diarrhoea. The GP considers diverticulitis as a potential cause and orders for a point-of-care CRP test, yielding a result of 47 mg/L. The GP has access to the full electronic healthcare data covering more than 20 years of data, including all medical notes, also from a previous episode of uncomplicated diverticulitis 8 years ago and recurrent irritable bowel syndrome complaints. Mrs. Sparrow currently wears a smartwatch, and – prior to this consultation – she already uploaded data on her pulse and temperature from the week prior to this consultation to her personal EHR-environment. Given that Mrs. Sparrow – unlike her previous episode of diverticulitis – now has concurrent medical problems, including poorly controlled diabetes, the GP is in doubt whether she should refer Mrs. Sparrow to the Emergency Department. Imagine how a medical Gen AI model including her full medical record and smartwatch data, as well as recent guidelines and literature might help sketch individual disease trajectories and the related management options for Mrs. Sparrow. Informed by this Gen AI application and augmented by the GP’s own intuitive and contextual knowledge, a much more personalised management approach can be discussed with the patient.

## What is needed to effectively stimulate the development of Generative AI in general practice?

It is timely to act as academic departments of general practice and take the lead in evaluating the development and implementation of Gen AI based innovations in general practice. In many cases these efforts potentially benefit from public–private partnerships, fostering each other’s competencies and qualities. Moreover, there is a pressing need to engage with the primary care community on how GPs, their staff, and patients perceive the benefits but also existing policy frameworks and the potential ethical, moral and legal challenges of Gen AI in primary care. Gen AI has the potential to change the roles and responsibilities of GPs. Consequently, its clinical use raises questions about accountability, expertise and responsibility for the clinical actions recommended [24]. Furthermore – similar to traditional AI technologies – the quality and diversity of the training set will determine how well its outputs are. Strategies to mitigate such so-called algorithmic bias – like ensuring diverse training sets and algorithmic audits and keeping humans-in-the-loop during both the development and testing phase – are crucial to ensure that these systems to not unintendedly increase health disparities [25]. In addition, it is important to acknowledge practical difficulties in relation to data collection, usage, storing and sharing, notably across borders. This is a highly contested area, requiring extra precaution given the sensitivity of medical data. In that respect, the recent publication and start of the transition phase towards application of the European Health Data Space Regulation will for sure help improve the sharing and trustworthy (re-)use of healthcare data across the European Union also for research, including for Gen AI [26].

Nevertheless, all these aspects clearly call for primary care leadership. If we don’t take this lead now, primary care risks being bypassed by private or commercial initiatives, which may not adequately consider the primary care perspective as well as address these ethical and legal implications. Even more so because the rapid emergence of Gen AI may shepherd valuable changes and improvements to primary care service and quality. Expectations may be overrated, and the reality may not match the current ‘hype’.

Thus, for primary care to be in the driving seat, what should be the research agenda that supports Gen AI efforts focussing on primary care?

First, agenda setting and prioritisation. It is vital for the GP community to be closely engaged with setting priorities. Part of the urgency is that multiple solutions are currently being developed and implemented without deep primary care expertise. It is critical that we avoid some of the problems we have seen with other technology implementations (e.g. electronic health records), by engaging GPs in agenda and priority setting. Participation of the broader international GP community is needed, as some Gen AI solutions may apply to multiple systems and countries (e.g. ‘voice-to-notes’ recording), whereas others are likely highly unique to a few systems (e.g. referral guidelines). While the pace of AI research and investment has moved rapidly, so far there have been few prioritisation efforts involving the pan-European GP community. A dedicated group of interested primary care researchers was formed during our workshop that aims to build such a research agenda to be discussed further during the WONCA 2025 World conference, in close collaboration with other relevant stakeholders like patient advocacy groups, data scientists, and ethicists.

Second, defining among the GP research community what evidence needs to be created. Gen AI tools are extremely broad in their aims, roles, types of data used, as well as their projected outcomes and possible adverse effects. Therefore, there is no ‘one-size-fits-all’ mandate for research to guide implementation. Rather, during agenda setting and prioritisation, we suggest using a structured classification scheme to link specific tools to the purposes it strives for: ‘only’ administrative support, patient or physician education, or optimising clinical decision-making. Subsequently, such classification can then guide the research needs and tailor study designs accordingly. Here, several existing technology implementation frameworks can be used, adapted to the Gen AI tool under scrutiny. For instance, for some Gen AI tools there may be little need for rigorous interventional research, but rather a focus on prospective evaluation of an optimal user experience after implementation etc (e.g. for ‘voice-to-notes’ systems). Appropriate evaluation of such tools will only need a focus on the usability experience, for instance using process evaluation study designs. For other Gen AI tools however – e.g. the diagnostic and therapeutic CDM tools where patient safety is at stake – comparative clinical intervention studies are needed with appropriate designs including pragmatic randomised clinical trials [27,28]. GP researchers can guide this calibration by being involved in drafting the right research protocols, choosing the right patient setting (clinical domain) and finally selecting the right interventions and their appropriate clinical outcomes. There is urgency, as much of what we describe is already under construction, and some applications have already been approved by regulators and have reached the market, notably sometimes without proper evaluation or with mixed results. This was, for example, recently shown in a vignette-based study comparing a Gen AI model (in this case ChatGPT) with experienced real physicians using complex primary care cases from Family Medicine clinics in Sweden [29]. Indeed, Swedish GPs performed better in these complex primary care cases, highlighting the need for rigorous development and evaluation of these novel Gen AI tools in primary care. Again, not every AI application needs to be evaluated in a randomised clinical trial. However, all AI applications which aim to improve individual patient care, such as those aiming at improved diagnostic and therapeutic CDM, may directly impact quality and safety. Their implementation needs to be based on rigorous scientific evaluation [30].

Third, we need to define who will do what in the development process. The private sector is investing deeply in various Gen AI tools (not all focussed on health specifically), with the expectation that at least some will be implemented (and improve care delivery, quality efficiency, etc.) and provide return on this investment. Successful development, testing and eventual implementation involves multiple partners, including GPs if the novel tool is specifically geared towards primary care. However, this cannot come from individual GPs alone, true engagement can only occur if our national academies, funding agencies and health authorities also support these efforts, linked to patient advocacy groups and interdisciplinary experts.

Finally, we need access to relevant data. AI requires the use of large datasets. For AI development in general practice, we therefore need (inter)national collaboration to create large international databases of routine primary care data, make them accessible and ensure they meet privacy and legal conditions. That will not only facilitate scientific evaluation of Gen AI based innovation but also leave room for detailing country- or culture- specific differences [31].

## Opportunities and future challenges

To conclude, Gen AI is rapidly entering society and thus also the realm of our clinical consultation rooms in general practice. Based upon feedback from our internationally attended WONCA Europe conference workshop we identify three priorities for AI in primary care: i) reducing administrative burden, ii) teaching and education, and iii) clinical-decision support. These priorities all require different elements of GP-involvement and rigorousness of scientific evaluations. Nevertheless, they all require a form of GP leadership and embracement of our academic departments of general practice. This is probably most so for Gen AI solutions that focus on improving patient outcomes and/or healthcare delivery by augmenting clinical decision-making. We believe our general practice community should take a central role in these new developments. Some of the opportunities and challenges that then need to be faced are summarised in Box 2. During the WONCA meeting participants suggested a broad international follow-up initiative to draw a research agenda for AI driven innovation in primary care.

Box 2. Opportunities and future challenges of Generative AI for general practice

Opportunities

Implementation of new Generative AI tools can lower the administrative burden in primary care, thereby freeing up time for patient related tasks.

Generative AI tools can serve as an educational assistant providing real-time, up-to-date and individualised learning plans for GPs and GP trainees.

Generative AI tools can help GPs addressing the increasing complexity of care through the development of clinical decision support systems and by increasing the access to more sophisticated diagnostic facilities in primary care.

Future challenges

The ethical implications of Generative AI models on clinical care are not yet fully elucidated; these implications include, among others, a changing perspective on roles and responsibilities of GPs on how to interpret Generative AI output and risks of algorithmic bias that potentially increases health disparities.

Data ownership, data access and privacy related topics can lead to distrust in society on who has access to personal medical data, notably in the realm of public–private collaborations.

The current hopes and hypes of Generative AI models can falsely lead to an overestimated believe of the trustworthiness of its output and thereby jeopardising patient safety, in particular if a human ‘checks and balances’ approach is ignored.
